# Managing Labour in Women with COVID-19

**DOI:** 10.3390/jcm12123980

**Published:** 2023-06-12

**Authors:** Victor Ngozi Chilaka, Osric Navti, Albert Opoku, Gbemisola O. Okunoye, Isaac Babarinsa, Olusegun Abiodun Odukoya, Abdulmalik Bako, Abdul Kareem Pullatttayl Sulaiman, Manoj Mohan

**Affiliations:** 1 Hamad Medical Corporation Qatar, Doha 3050, Qatar; 2 Weill Cornell Medicine Doha, Doha P.O. Box 24811, Qatar; 3 Sidra Medicine Qatar, Doha P.O. Box 26999, Qatar; 4 University of Health & Allied Sciences, Ho, Ghana; 5 Qatar University College of Medicine, Doha P.O. Box 2713, Qatar; 6 Queen’s University, Kingston, ON K7L 3N6, Canada; 7 Aster Hospital, Doha 24450, Qatar

**Keywords:** COVID-19, antenatal care, labour, anaesthesia, analgesia, pregnant labouring women delivery and breastfeeding

## Abstract

Since first reported in December 2019 in Wuhan, China, COVID-19 caused by Severe Acute Respiratory Syndrome (SARS) Corona virus2 (SARS CoV-2) quickly spread to become a pandemic that has caused significant morbidity and mortality. The rapidity of the spread of the virus and the high mortality at the outset threatened to overwhelm health systems worldwide, and, indeed, this significantly impacted maternal health, especially since there was minimal experience to draw from. Experience with Covid 19 has grown exponentially as the unique needs of pregnant and labouring women with COVID-19 infection have become more evident. Managing COVID-19 parturients requires a multidisciplinary team consisting of anaesthesiologists, obstetricians, neonatologists, nursing staff, critical care staff, infectious disease and infection control experts. There should be a clear policy on triaging patients depending on the severity of their condition and the stage of labour. Those at high risk of respiratory failure should be managed in a tertiary referral centre with facilities for intensive care and assisted respiration. Staff and patients in delivery suites and operating rooms should be protected by enforcing infection protection principles such as offering dedicated rooms and theatres to SARS CoV-2 positive patients and using personal protective equipment. All hospital staff must be trained in infection control measures which should be updated regularly. Breastfeeding and care of the new-born must be part of the healthcare package offered to COVID-19 parturient mothers.

## 1. Introduction

In December 2019, the world learned of the first case of a patient infected with atypical pneumonia caused by the 2019 novel coronal virus (severe acute respiratory syndrome coronavirus-2 or SARS-CoV-2) in Wuhan, China [1]. The infection spread rapidly, and by mid-March 2020, more than 190 countries had reported cases prompting the WHO to declare it a pandemic. In February 2020, the World Health Organization designated the disease as COVID-19, which stands for coronavirus disease 2019 [1,2]. Over half a billion COVID-19 infections worldwide and over 6 million deaths have so far been reported [2].

As is typical with viruses in this genre, mutations frequently lead to the emergence of new strains. There are currently five strains of the SARS-CoV-2 virus that are of concern: the Alpha, Beta, Gamma, Delta and Omicron variants [3]. These variants have specific traits, including increased transmissibility and a tendency to cause more severe disease for some. While the Delta and Alpha variants seem to be associated with more severe disease, the Omicron variant is associated with less severe illness but is more infectious. Globally as of May 2023, WHO is currently monitoring two variants of interest (VOIs), XBB.1.5 and XBB.1.16, along with seven variants under monitoring (VUMs) and their descendent lineages [4]. Currently, available information does not suggest that XBB.1.5 has additional public health risks relative to the other presently circulating Omicron descendent lineages [5].

The rapidity of the spread of the virus and the relatively high mortality of the initial variants threatened to overwhelm health systems worldwide. It impacted maternal health significantly with minimal experience to draw from. Because of this, various nations applied untested drugs, techniques and means to control the spread, including lockdowns, which resulted in isolation and poor attendance and, sometimes, neglected ante-natal care. Using some untested but supposedly life-saving medications on pregnant and labouring women severely challenged the ethics and principles of the medical profession.

Experience has since been growing, and the unique needs of pregnant and labouring women with COVID-19 are becoming more evident. They must be considered while making treatment policies and preparing response plans to COVID-19.

The impact of vaccination and the emergence of supposedly less virulent strains has undoubtedly affected the course of COVID-19. However, it is still likely to continue to cause adverse maternal and neonatal outcomes, especially in those who are unvaccinated [3].

In this review, we will examine the impact of COVID-19 on the management of labour.

## 2. COVID and Pregnancy

Current evidence suggests that pregnancy does not increase susceptibility to SARS-CoV-2 infection but appears to worsen the clinical course of COVID-19 (e.g., increased risks of intensive care unit [ICU] admission, need for mechanical ventilation and ventilatory support, and death) compared with non-pregnant females of the same age, especially in those who are symptomatic [6,7,8,9]. The risk of an exacerbation is greatest in the third trimester. Risk factors for severe disease and death in pregnancy include older age (especially ≥35 years), obesity, pre-existing medical co-morbidities (particularly hypertension, diabetes, or more than one co-morbidity), and being unvaccinated [9,10,11,12]. Other risk factors associated with being infected and hospitalised with COVID-19 include Black and Asian minority ethnic backgrounds, living in socioeconomically deprived areas, and working in healthcare or other public-facing occupations [10]. Recent evidence suggests that pregnancy-related risks can be reasonably minimised or mitigated by standard preventive measures, including vaccination [10].

## 3. Effects of COVID on Pregnancy and Labour

One of the most important reports on the effect of COVID-19 infection in pregnancy was that by Villar et al. (2021) [13]. They undertook a case-controlled retrospective study of 706 pregnant women diagnosed with COVID-19 and 1424 pregnant women without COVID-19 [13]. The women with COVID-19 diagnosis had a higher risk for preeclampsia/eclampsia (relative risk [RR], 1.76; 95% CI, 1.27–2.43), severe infections (RR, 3.38; 95% CI, 1.63–7.01), intensive care unit admission (RR, 5.04; 95% CI, 3.13–8.10), maternal mortality (RR, 22.3; 95% CI, 2.88–172), preterm birth (RR, 1.59; 95% CI, 1.30–1.94) and medically indicated preterm birth (RR, 1.97; 95% CI, 1.56–2.51). It was also notable that fever and shortness of breath for any duration were associated with an increased risk of severe maternal complications. However, asymptomatic women with COVID-19 remained at higher risk for maternal morbidity (RR, 1.24; 95% CI, 1.00–1.54) and preeclampsia (RR, 1.63; 95% CI, 1.01–2.63).

In-utero transmission of COVID-19 is rare, and there is no evidence of an increase in spontaneous miscarriage, fetal congenital structural anomalies or adverse perinatal outcomes. However, the risk of spontaneous and iatrogenic preterm birth is increased [9,14].

## 4. Effects of Pregnancy and Labour on COVID

A lot of factors determine the severity of COVID-19 disease in pregnancy, and one of these factors may be the hyperinflammatory immune response which may be responsible for multi-organ damage. It has been noted that ferritin and IL-6 levels were lower in a survivor compared to a non-survivor group, as was the finding of higher neutrophil-lymphocyte ratio, lower percentages of basophils, eosinophils, and monocytes in severely-ill subjects [15].

### 4.1. Breathing Problems

Although the predominant features of SARS-CoV-2 infection, whether in pregnancy or not, are respiratory, it should be remembered that normal respiratory changes in pregnancy may mimic these symptoms. Constantly monitoring oxygen saturation is an essential aspect of care for the COVID-19 patient during pregnancy and labour.

### 4.2. Pushing

Women with a cough or breathing difficulties, or those who feel unwell, should be closely monitored for their oxygen saturation and other vital signs, more so when they are in labour. There should be a low threshold for supplementary oxygen [16].

The Valsalva manoeuvre in the second stage of labour is an aerosol-generating event, and healthcare providers must take measures to protect themselves and their birthing partners. Parturient women with worsening respiratory symptoms or exhibiting exhaustion with the bearing down efforts should be offered assisted delivery [16,17].

### 4.3. Temperature Swings

It was initially believed that fever is the most frequently reported manifestation of COVID-19. It was, therefore, a critical element of screening for COVID-19. However, it now appears more common in patients with severe or progressive disease [17]. In the report of the first 1000 SARS-CoV-2 patients managed in New York in 2020 [18], fever was almost as common as cough amongst the presenting symptoms (728/1000 vs. 732/1000), respectively. Control of temperature swings is thus essential in managing labour in these patients.

## 5. Screening, Investigations and Diagnosis

The occurrence of asymptomatic COVID-19 has led to the recommendation for universal COVID-19 testing for all elective and emergency admission of pregnant women in labour and postpartum. A policy of universal pre-admission testing enables the use of the pregnant woman’s COVID-19 status to determine the appropriate place of care. It also helps in the timely implementation of isolation procedures as well as mobilisation of the required personal protective equipment (PPE), ultimately reducing the risk of nosocomial covid infection [19,20].

Symptoms of COVID-19 in pregnant women typically include fever (temperature ≥ 37.8 °C) in association with other symptoms, including breathing difficulty, shortness of breath, sore throat, gastrointestinal symptoms and new onset of loss of smell or taste [21,22,23]. Symptomatic women should have a nasopharyngeal swab for reverse transcriptase polymerase chain reaction(RT-PCR) testing for laboratory confirmation of COVID-19 [24]. The need for prompt availability of results crucial for symptomatic pregnant women presenting in labour has led to the introduction of point-of-care testing such as the rapid antigen test (RAT). Pregnant women who on admission are RAT positive should have a confirmatory RT-PCR test with an added report on the cycle threshold (Ct) value. The Ct value is derived from the RT-PCR result as a proxy for viral load. Although there is a lack of standardisation of RT-PCR Ct value, it has been used in clinical settings as a diagnostic adjunct to triaging patients based on the likely level of infectivity [25]. Most patients tolerate the nasopharyngeal swab, but care should be taken in those with abnormal nasal septum anatomy from surgery. Adequate staff training with quality control measures is vital in ensuring proper sampling technique and integrity of the entire diagnostic process. A recent Cochrane review supported the use and performance of the RAT as a diagnostic tool for COVID-19 [25]. The authors concluded that Antigen tests vary in sensitivity. In people with signs and symptoms of COVID-19, sensitivities are highest in the first week of illness when viral loads are higher. Assays that meet appropriate performance standards, such as those set by WHO, could replace laboratory-based RT-PCR when immediate decisions about patient care must be made or where RT-PCR cannot be delivered promptly. However, they are more suitable for use as triage to RT-PCR testing. The variable sensitivity of antigen tests means that people who test negative may still be infected.

Pregnant women with negative RT-PCR test but who develop worsening respiratory symptoms suggestive of COVID-19 should be offered a chest X-ray or CT scan as up to 60% of infected patients may have positive signs on CT scan consistent with COVID-19 prior to the initial positive RT-PCR test. The typical CT appearances of COVID-19 lungs include consolidation (50%) and ground glass or opacity (46%) [26].

Symptomatic pregnant women, especially those at term with co-morbidities, should have additional ancillary laboratory testing for illness severity assessment. The initial tests should include a complete blood count, complete metabolic panel (CMP), arterial blood gas, and a chest X-ray. Additional tests should be carried out if baseline tests are reported as abnormal. Further tests include electrocardiograph (ECG), coagulation profile, C reactive protein (CRP), ferritin, D-dimer, lactate dehydrogenase (LDH), B-type natriuretic peptide (BNP) as well as Troponin [21]. (Box 1).

Box 1 **Investigations of COVID-19 patients.**

Basic Investigations:

Complete Blood Count (CBC)—thrombocytopenia and lymphopenia are associated with severe disease

Complete Metabolic Panel (CMP)—to assess multisystem effects and complications like drug induced hepatitis

Arterial blood gas—assess hypoxemia

Further tests:

* CRP, Procalcitonin, ** LDH, *** CK, Ferritin—assess inflammatory response

Coagulation profile—assess for ^§^ DIC and potential risk of obstetric haemorrhage, D-dimer not useful for diagnosis of ^±^ VTE in pregnancy

Lactate dehydrogenase (LDH) to access the level of haemolysis and tissue injury by COVID

B-type natriuretic peptide (BNP) as an indicator of clinical severity of COVID

Troponin—assess for cardiac involvement.

Electrocardiograph (ECG),

Chest X-ray

Abbreviations:

* CRP—C-reactive protein. ** LDH—Lactate dehydrogenase, *** CK—creatine kinase. ^§^ DIC—disseminated intravascular coagulopathy. ^±^ VTE—venous thrombo-embolism

## 6. Care of the COVID Positive Mothers

### 6.1. Principles for Caring for COVID-19-Positive Mothers in Labour

Care of COVID positive labouring women should be multidisciplinary, involving obstetricians, physicians (respiratory/infectious disease/intensivists/critical care), anaesthesiologists, neonatologists, and nursing and midwifery specialities.

The management guidelines for women with COVID-19 in labour have evolved as more experience and research have become available. The risk factors for severe/critical COVID-19 infection in pregnancy must be borne in mind [6,27,28,29,30].

Severity classification is an essential part of management planning and is based on clinical, biochemical and radiological parameters. One good example of such classification is the US NIH Classification which categorises them as Asymptomatic or Pre-symptomatic Infection, Mild Illness, Moderate Illness, Severe Illness, and Critical Illness [30].

### 6.2. Out-Patient Management

**Figure 1:**
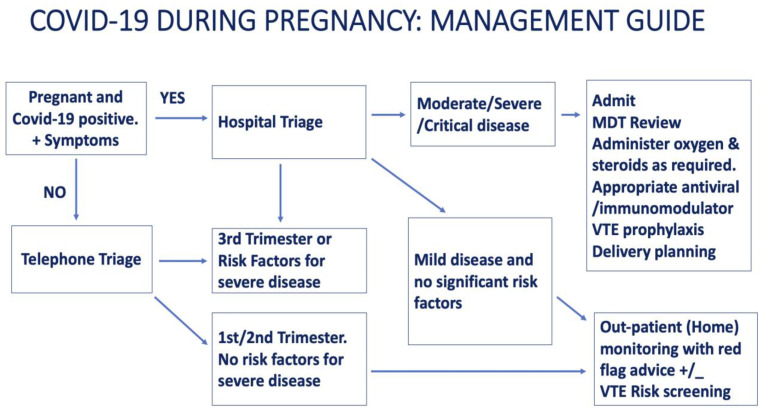
Flowchart for the management of COVID-19 in pregnancy.

Asymptomatic and mildly symptomatic SARS-CoV-2 positive pregnant women can be managed at home with red flag advice until labour is established. They should monitor their temperature and, importantly, oxygen saturation at home. Telephone/Video triage and consultations, where available, enable out-patient management of these patients with hospital admission when they are most likely in established labour or develop obstetric and other emergencies. As the disease severity can evolve rapidly and unpredictably, these patients should have clear parameters for attending emergency services. Such parameters include the onset of strong and regular contractions or, on the other hand, fever not resolving with antipyretics, increasing shortness of breath, difficulty speaking complete sentences, and confusion/changes in the mental state [28,31]. There are algorithms produced by various national and international professional bodies to guide the management of COVID-19 during pregnancy. One such algorithm is shown in Figure 1.

### 6.3. In-Patient Management

COVID-19 pregnant women with moderate/severe symptoms should be seen and assessed in person. There should be a local policy directing symptomatic women to appropriate service locations such as dedicated COVID facilities, obstetric triage or other emergency locations to minimise contact with other patients. Depending on the local protocols, these women must be assessed by a multidisciplinary team. Assessment should determine the severity of the disease, the stage of labour, and the subsequent level of care required until delivery.

The laboratory investigations listed in Box 1 should be performed on admission and repeated as clinically indicated.

Other laboratory investigations that may be indicated include those that aim to exclude differential diagnoses and assess the risk of severe disease, e.g., viral hepatitis screen, influenza panel, and HIV. Though pyrexia is a common feature of COVID-19, pregnant women presenting with pyrexia should have thorough clinical and microbiological assessments for other possible causes, including a full septic screen.

Pregnant women in labour should have the same clinical assessments and interventions as the non-pregnant population. Investigations such as chest X-rays should not be restricted based on perceived risks to the fetus. Chest ultrasound has been recommended as a safe and feasible alternative to chest X-rays to assess for COVID-19 pneumonia. It can be performed alongside obstetric ultrasounds with no concern about radiation exposure [32,33]. Other investigations should be performed as clinically indicated and targeted to the differential diagnoses and complications, including Chest CT scans/CTPA/ECG/echocardiograms for women with suspected pulmonary embolism (PE) and myocardial infarctions [31,34].

Most patients with SARS-CoV-2 do not have pneumonia. Those with pneumonia are likely to have other co-morbidities. The radiological findings of ground glass appearance of the lungs with diffuse alveolar destruction are almost diagnostic [15]. The acute respiratory distress syndrome associated with COVID-19 is unique [15,35]. The use of oxygen supplementation, corticosteroids, non-invasive positive pressure ventilation (to delay the need for endotracheal intubation) and extracorporeal membrane oxygenation (ECMO) has saved lives [35]. The additional requirements of the labouring woman will put a higher demand on her systems.

Monitoring of the patient must include frequent measurements of blood pressure (BP), pulse rate, respiratory rate, oxygen saturation and temperature. The disease severity and patient symptoms will determine the frequency of these observations, ranging from four hourly in mild/moderate disease to hourly or continuous in severe/critical disease.

### 6.4. Fetal Monitoring

There are reports linking COVID-19 with impaired placental function and fetal growth restriction [13,28].

Acute fetal compromise, non-reassuring fetal heart rate patterns and increased perinatal mortality are associated with severe COVID-19 [13,28]. Women admitted with severe disease should have a fetal assessment and neonatal consultation as part of their multidisciplinary care. They should have scheduled regular/continuous fetal monitoring, which can be challenging in the critical care setting but must be continued throughout labour. Steroids for fetal lung maturation should be given, and magnesium sulphate (MgSO_4_) for neuroprotection should be considered for those in preterm labour less than 34 weeks. Potential explanations for the fetal compromise include maternal pyrexia and hypoxemia and COVID-19-specific pathological changes in the placenta, dubbed COVID-19 placentitis [36].

### 6.5. Fluid Management

Fluid management must ensure a neutral balance, as overload can worsen respiratory compromise. Strict input and output charts should be maintained to guide fluid balance. Fluid boluses may be instituted instead of continuous infusions where fluid resuscitation is required.

### 6.6. Supplemental Oxygen

Oxygen saturations should be maintained above 94% for optimum maternal and fetal perfusion. Oxygen supplementation should be escalated from nasal cannula, non-rebreathe oxygen masks, high flow oxygen masks, and CPAP to mechanical ventilation and ECMO as required.

A local protocol to recognise clinical deterioration and escalation of care is essential. With increasing oxygen requirements, arterial blood gases help to better determine the level of hypoxemia and early involvement of intensivists is recommended. It is recognised that pregnant women often compensate for serious illness until late and that the respiratory rate and oxygen saturation parameters are the same for pregnant and non-pregnant patients, which can lead to late recognition. Late recognition of the clinically deteriorating parturient can lead to severe maternal morbidity and mortality. Early resort to life-saving interventions like intubation, mechanical ventilation, and ECMO should be applied with the same qualifying criteria in labouring, pregnant and non-pregnant women [31,34,37,38]. Furthermore, urgent delivery of the baby should be part of this consideration.

Pregnant women with COVID-19 in labour with increasing oxygen requirements should have a multidisciplinary review and discussion about the benefits of expedited delivery to ease the respiratory burden and improve oxygen delivery.

### 6.7. Proning

The nursing of pregnant women with COVID-19 and moderate to severe hypoxaemia, respiratory failure, and ARDS in the prone position, hitherto thought to be detrimental, has been shown to reduce the need for and the duration of intubation and mechanical ventilation and mortality [39,40]. Consideration should be given to appropriate padding to offset the gravid uterus and prevent aortocaval compression [41]. Proning can be challenging in the third trimester and labour. Continuous fetal monitoring can be equally challenging during proning. The benefits of delivery should be discussed within the multidisciplinary team and with the woman/relatives as appropriate [41,42].

### 6.8. Thromboprophylaxis

Venous thromboembolism (VTE) is a well-recognised pregnancy complication and COVID-19 infection. All pregnant women with COVID-19 presenting in labour should be risk assessed for VTE using risk scores, e.g., RCOG Guideline Reducing the Risk of Venous Thromboembolism during Pregnancy and the Puerperium (2015) [43], and treated accordingly. Appropriate thromboprophylaxis should be offered for at least ten days or until discharge. There is no consensus on whether to give the routine weight-based prophylactic or a higher prophylactic dose [28,30,43].

### 6.9. Pharmacotherapy

Several medications have been used on patients with COVID-19, initially extrapolated from other therapeutic uses with emergency use authorisations. Initially, pregnant women were excluded from research into drugs and other life-saving interventions such as vaccination, but some large-scale drug trials involving pregnant women (e.g., RECOVERY [44], SOLIDARITY [45]); have led to the current recommendations. The WHO published a Living Guideline on COVID-19 therapeutics and recommended that pregnant women with severe/critical disease should not be denied any potentially beneficial therapy for fear of unknown detrimental fetal effects, and labour should be no different [46].

The RECOVERY Trial [44] showed that steroids, especially dexamethasone 6 mg daily, improved clinical outcomes and minimised critical care requirement/duration and maternal mortality. It is recommended as a 10-day course or until discharge in severe COVID-19, with clinical modification in critical disease. As there is some concern about adverse neonatal outcomes with prolonged courses of dexamethasone and other fluorinated steroids, prednisolone 40 mg daily/methyl-prednisolone 32 mg daily or iv hydrocortisone 100 mg daily can be used as substitutes. In women who require steroids for fetal lung maturation, Dexamethasone/Betamethasone 12 mg 24 h apart is used for the first 48 h and then switched to a non-fluorinated steroid to complete the course [28,47]. Treatment should not be discontinued in labour.

Tocilizumab and Sarilumab (interleukin-6 receptor antagonists)- improve outcomes in pregnant women with severe/critical disease, especially with hypoxia and systemic inflammatory response (the so-called cytokine storm) [44]. There is not much safety data on pregnancy for Sarilumab. However, the current recommendations are to use it if Tocilizumab is unavailable for patients meeting the criteria (severe/critical illness, CRP > 75). As these drugs affect immunity, women exposed to them should be advised about the increased risk of unusual infections and neonates exposed in-utero should defer receiving live vaccines for at least six (6) months [34,44,48].

Neutralising monoclonal antibodies (nMABs) are recommended for pregnant COVID-19-positive patients who are very high risk/immunocompromised and unable to mount an antibody response to COVID-19 (negative Anti-Spike antibodies), following discussion at the MDT [28,46].

Remdesivir is an intravenous antiviral agent currently conditionally recommended for non-hospitalised patients with a risk of severe disease (patients on oxygen but not mechanical ventilation) or those deteriorating or not improving. There are limited safety data on pregnancy and breastfeeding, and it should only be used where the benefit outweighs the risk [28,46].

Aspirin has not been shown to significantly improve the risk of mechanical ventilation or 28-day mortality in women with severe COVID-19. Still, it can result in a slight improvement in the chances of being discharged alive. Women on aspirin for preeclampsia or IUGR prophylaxis should continue with medication unless there is severe thrombocytopenia, with an attendant risk of obstetric haemorrhage [28,49].

Women on antiviral medications should continue treatment through labour, which is not a contraindication to instituting appropriate antiviral therapy if indicated. Current recommendations are not to use Lopinavir-ritonavir (Kaletra), Azithromycin, Hydroxychloroquine, and Ivermectin in COVID-19 as they are not beneficial.

### 6.10. Timing and Place for Delivery

Delivery decisions should be based on standard obstetric practice and indications in the main, except in special circumstances, as discussed below. Local policies will determine the place of delivery. With different waves of the pandemic, arrangements have been made with designated COVID hospitals and labour units and the allocation of specific wings/rooms within standard labour wards for COVID-positive women.

Depending on the community infection and vaccination rate, healthcare facilities will have different screening protocols for women being admitted electively for induction of labour and Caesarean delivery. These range from clinical/symptom/positive contact-based telephone triage to universal screening with nCov-19 RT PCR for all elective admissions performed 24–48 h before admission. Emergency admissions are similarly screened with the Rapid Antigen Test +/− PCR based on local policy and availability.

Whether birth partners can accompany labouring COVID-19 women should be determined by local rates of community infection and infection control policies. Where birth partners are allowed, they will need to be screened by clinical symptoms/contact with Covid positive patient, temperature and RAT at presentation or PCR done 24 h before planned admissions for elective inductions or Caesarean delivery. Birth partners should wear appropriate PPE (face masks/covering) and stay with the woman in the allocated delivery/postnatal rooms.

Local infection control policies will guide the management of SARS-CoV2 infection control and prevention. In general, it is advised that healthcare facilities practice contact and droplet protection for COVID-19 and airborne protection, including using negative pressure rooms for aerosol-generating procedures, including intubation for general anaesthesia. Attendant staff with COVID-19 pregnant women should wear appropriate PPE, including FFP3/N95 masks and goggles/face shields.

## 7. Management of Delivery

### 7.1. Asymptomatic/Mildly Symptomatic Women

Asymptomatic or mildly symptomatic COVID-19-positive women can have their labour and delivery as appropriate in a labour suite, low-risk birthing centre and also at home, depending on local arrangements and availability of midwifery support. There is insufficient evidence for or against water births in these women, the main concerns being maintaining the integrity of PPE equipment on birth attendants and the unknown risks of viral transmission in water. Decisions for water birth should be discussed between the woman and her care team [28,30,34].

### 7.2. Women with Moderate and Severe Disease

COVID-positive women with moderate to severe disease are advised to deliver in the hospital as they need close monitoring of both the mother and fetus [30]. Women with moderate disease should be assessed in person to determine whether they need hospital admission. If they do, delivery planning should be part of the multidisciplinary discussion. Women between 24–34 weeks’ gestation should receive steroids for fetal lung maturation as part of their management [17,30].

There should be a discussion within the clinical care team as to the benefits of deferring planned induction of labour/elective Caesarean deliveries in those with moderate disease near-term unless restricted by other obstetric considerations [30]. The benefits of avoiding the stress of labour and delivery and the spread of active infections to healthcare workers should be weighed against the potential risks of worsening disease severity in the mother and associated increased risk of perinatal morbidity/mortality [50,51].

On admission, those with moderate COVID-19 presenting in labour should have a senior obstetrician and anaesthetic assessment. Oxygen saturations should be kept above 94% with supplemental oxygen as required, and routine intrapartum observations should be undertaken alongside any other observations necessary for COVID-19 in the local protocol [30]. They should also have continuous electronic fetal monitoring as COVID-19 has been associated with higher incidences of acute fetal compromise, CTG abnormalities and perinatal mortality [38]. The application of fetal scalp electrodes is not contraindicated in women with COVID-19 [30,38].

Spontaneous preterm labour and premature rupture of membranes are thought to be associated with maternal pyrexia [27,52]. However, a significant proportion of preterm birth in those with COVID-19 is iatrogenic, with delivery aimed at improving maternal oxygenation. The mode of delivery should follow the standard obstetric practice [17,30]. Caesarean section should be undertaken for obstetric indications unless thought to be expedient to improve the maternal condition [30].

The diagnosis of preeclampsia in severe COVID-19 in labour may be challenging in some cases, especially as there is an overlap with the biochemical parameters of COVID-19 multi-system disorders, especially elevated liver transaminases and thrombocytopenia [53,54]. MgSO_4_ use for eclampsia prophylaxis should be monitored closely in those with respiratory compromise and renal impairment due to severe COVID-19, as the risk of magnesium toxicity may be increased [54]. Dose adjustments, frequent monitoring of serum magnesium levels and involvement of intensivists are recommended [30,55].

Women, especially those in the late second or third trimester with increasing oxygen demands requiring mechanical ventilation, should discuss the benefits of delivery to ease the respiratory burden and improve oxygen delivery and perinatal outcomes. Induction of labour has been safely performed in pregnant women with severe COVID-19 having mechanical ventilation; Caesarean sections have been performed safely in women on ECMO [34,56,57].

### 7.3. Mode of Delivery

COVID-19 should not affect the planned mode of delivery in most women who are asymptomatic or have mild/moderate disease, and delivery decisions should be based on standard obstetric practice [6,28].

COVID-19 is not an indication for Caesarean section. However, it is associated with a higher Caesarean section rate (about 50% versus <30% in the general population) in most reports on women with symptomatic and severe disease for maternal and fetal compromise [6,38]. Initial concerns about an increasing overall Caesarean section rate during COVID-19 have not been confirmed. For example, in some countries, infected women had a higher-than-expected rate of cesarean delivery (CD) [58]. In contrast, other studies showed that the overall Caesarean section rate was either unchanged compared to the pre-pandemic rate [59,60] or even lower [61,62]. Whether infection with COVID-19 at the time of delivery is an indication for Caesarean delivery is unknown [9,63] as there is no clear evidence of vertical transmission [64]. Nonetheless, COVID status alone seems to be a common indication for caesarean delivery, particularly in the early stages of the pandemic [65].

Consideration should be given to transferring severely or critically ill women in labour to tertiary centres with facilities for ECMO and other critical care interventions.

Assisted vaginal delivery should be performed for obstetric indications. It may be clinically indicated in selected cases with severe/critical COVID-19 to shorten the second stage and minimise maternal effort in those with respiratory compromise, especially if on mechanical ventilation [17,30].

The risk of postpartum haemorrhage has been noted to be increased in women with COVID-19 [66,67], and precautions should be taken to minimise this [30,68]. This may be associated with other known complications, e.g., thrombocytopenia/DIC [66,67].

### 7.4. Anaesthesia and Analgesia in Labour

All pregnant women with COVID-19 admitted in labour should have an anaesthetic review as part of their multidisciplinary care. All available options for labour analgesia should be offered as for non-COVID patients. Though there was initial concern about nitrous oxide usage and the potential of viral spread through the breathing tube/masks, individual patient use of tube/mask with a microbiological filter is a safe option. The use of nitrous oxide is not considered an aerosol-generating procedure [69,70].

There is also some concern about using parenteral opioids and the risk of respiratory suppression, especially in women with respiratory compromise [71,72]. The right patient selection with appropriate clinical review and close monitoring of respiratory rate and oxygen saturation should prevent any adverse outcomes. Parental opioids may be best avoided in patients with respiratory compromise.

Most obstetric anaesthetists recommend regional neuraxial blockade for labour analgesia in COVID positive parturients as it offers many benefits beyond analgesia, such as preventing pain-induced panting and screaming, which could be potential sources of droplet spread. It is also a viable option for emergency Caesarean section if required. Important considerations that can affect the use of neuraxial blockade include thrombocytopenia, recent thromboprophylaxis and maternal fever—all known features of COVID-19.

General anaesthesia (GA) for Caesarean section is considered an aerosol-generating procedure with potential droplet spread to healthcare workers in attendance. General anaesthesia in pregnant women is a high-risk procedure with concerns about the failure of intubation, aspiration and fetal respiratory suppression. With the added risks of SARS-CoV-2 exposure- obstetric anaesthetists advise that there should be local policies in place to minimise the associated risks, including [69,70].

- Preferable performing GA in a negative pressure room with airborne protection
- Performing GA only when necessary
- Having checklists to ensure all requirements are in place
- Wearing appropriate PPE
- Having the most experienced anaesthetist available to perform the intubation to ensure a first pass
- Use of video laryngoscopy where available
- Employing mechanisms to minimise coughing during extubation.
- Minimising the number of staff present in the theatre

Local arrangements should be in place for the safe transfer of COVID-19 mothers to theatres, and provisions made for potential delays from the donning of PPE by staff in cases of urgent Caesarean sections. Practice drills can improve the efficiency of this process.

## 8. Postpartum Care

In general, patients with suspected or confirmed SARS-CoV-2 infection should be isolated from uninfected patients and cared for according to standard infection control guidelines. The intensity of maternal monitoring in patients with COVID-19 depends on maternal status, with routine monitoring adequate for those with mild disease escalating to intensive care for those with more severe disease. Venous thromboembolism is indicated based on the routinely applied risk factors post-delivery. Symptomatic management of fever and pain is routine, as in any postnatal patient. The decision to discharge a patient with COVID-19 is generally the same as that for other conditions and depends on the need for hospital-level care and monitoring. All patients should be counselled on the warning symptoms that should prompt reevaluation by a telehealth or in-person visit, including emergency department evaluations. These include new onset of dyspnea, worsening dyspnea, dizziness, and mental status changes, such as confusion [73].

## 9. Perinatal Outcomes

There is inconclusive evidence that transplacental transmission of SARS-CoV-2019 occurs during pregnancy and through breast milk [74]. However, vertical transmission of COVID-19 has been documented with a rate of 1–2% [28,34]. Severe peripartum maternal disease, mechanical ventilation and maternal death are thought to increase the risk, which is unchanged by Caesarean section. Maternal vaccination and infection provide passive immunity to newborns. Delayed cord clamping, skin-to-skin contact, breastfeeding and rooming-in should be encouraged where possible [28,29,75].

Postnatal transmission of the virus through droplets can occur from mother to baby by close contact or by contact with an infected father, family members, carers, visitors, and health care staff [76].

## 10. Breastfeeding and Care of the New-Born

Breastfeeding promotes infant mental health and well-being and facilitates parent-baby bonding [77]. In vaccinated mothers, breastfeeding has the potential to offer passive immunity to the infant and may be protective against SARS-CoV2 [76,78]. The WHO has recommended exclusive breastfeeding of infants born to women with suspected or confirmed COVID-19 for at least the first six months while using infection prevention and precaution measures [79].

It is essential that breastfeeding is discussed with women and their families before delivery and after childbirth. Breastfeeding support should also be provided to women and their partners before and after birth, including information about how the partner can support the breastfeeding mother. This should be done regardless of their choice of method. Indeed, the value of the partner’s involvement and support should be explained and encouraged in keeping with the woman’s preferences about their involvement [80].

Women should also be given information about all other feeding methods before and after childbirth to support informed decision-making. Information could take the form of oral in-person discussion or written or digital information, including web-based information [76]. Antenatal and postnatal women and their families should be informed of recognising when their baby may be seriously ill and how to seek help as soon as possible.

## 11. Promoting and Supporting Breastfeeding

Asymptomatic women and those without severe COVID-19 symptoms should be supported to breastfeed while practising infection preventive measures. The WHO recommends that “a woman with COVID-19 should be supported to breastfeed safely, hold her newborn skin-to-skin and share a room with her baby” [79]. Mothers who are not severely ill should be allowed skin-to-skin contact with their infants and share a room while applying appropriate infection prevention and control measures [79]. Infants of severely sick women with COVID-19 can still be breastfed by their family and or health care practitioners using formula or expressed breast milk while applying appropriate infection prevention control measures [81,82,83].

Breastfeeding by COVID positive women should be based on the approach of shared decision with the woman and their immediate family. Promoting breastfeeding should respect women’s personal space, cultural influences, preferences, and previous infant feeding experiences [80].

## 12. Limiting Postnatal Transmission

Baby Friendly Hospital Initiative was introduced in 1991 to promote, protect and support breastfeeding. The WHO and UNICEF launched an initiative that includes ten steps to successful breastfeeding to help encourage healthcare facilities to support and promote breastfeeding [84]. Early skin-to-skin connection with face-to-face interaction should be encouraged to promote emotional attachment and bonding. Infants of women with COVID-19 who are breastfeeding or well enough to breastfeed should not be separated from their mothers [85]. Rooming-in should be facilitated whether the mother or baby has suspected, probable or confirmed COVID-19 [80].

Infants being breastfed should be observed for any symptoms or signs of illness, and parents should be advised on how to seek help when they suspect their child may be unwell. Baby scoring systems can be considered and used to determine when to seek help [80].

## 13. Conclusions

COVID-19 infection continues to be a serious health problem worldwide, although the course has been modified by vaccinations and the emergence of less virulent variants. Infections of parturients put them at significant risk of morbidity and mortality risk. They should be cared for by a multidisciplinary team consisting of anaesthetists, obstetricians, neonatologists, nursing staff, critical care experts, infectious disease experts, and infection control experts. Serious infection prevention procedures must be part of the plan and be applied to protect the workforce and other patients.

Breastfeeding requires careful planning and must not be denied to women wishing to do so but must be done with the application of infection control measures to protect the newborn, hospital staff and family members.

## Data Availability

Not applicable.
